# Model-based analysis of chromatin interactions from dCas9-Based CAPTURE-3C-seq

**DOI:** 10.1371/journal.pone.0236666

**Published:** 2020-07-31

**Authors:** Yong Chen, Yunfei Wang, Xin Liu, Jian Xu, Michael Q. Zhang

**Affiliations:** 1 Department of Molecular and Cellular Biosciences, Rowan University, Glassboro, New Jersey, United States of America; 2 Department of Biological Sciences, Center for Systems Biology, University of Texas, Dallas, Richardson, Texas, United States of America; 3 Department of Melanoma Medical Oncology, University of Texas MD Anderson Cancer Center, Houston, Texas, United States of America; 4 Children’s Medical Center Research Institute, Department of Pediatrics, University of Texas Southwestern Medical Center, Dallas, Texas, United States of America; 5 MOE Key Laboratory of Bioinformatics, Tsinghua University, Beijing, China; 6 Bioinformatics Division and Center for Synthetic & Systems Biology, BNRist, Tsinghua University, Beijing, China; 7 Department of Automation, Tsinghua University, Beijing, China; Università degli Studi di Milano, ITALY

## Abstract

Deciphering long-range chromatin interactions is critical for understanding temporal and tissue-specific gene expression regulated by *cis*- and *trans*-acting factors. By combining the chromosome conformation capture (3C) and biotinylated dCas9 system, we previously established a method CAPTURE-3C-seq to unbiasedly identify high-resolution and locus-specific long-range DNA interactions. Here we present the statistical model and a flexible pipeline, C3S, for analysing CAPTURE-3C-seq or similar experimental data from raw sequencing reads to significantly interacting chromatin loci. C3S provides all steps for data processing, quality control and result illustration. It can automatically define the bin size based on the binding peak of the dCas9-targeted regions. Furthermore, it supports the analysis of intra- and inter-chromosomal interactions for different mammalian cell types. We successfully applied C3S across multiple datasets in human K562 cells and mouse embryonic stem cells (mESC) for detecting known and new chromatin interactions at multiple scales. Integrative and topological analysis of the interacted loci at the human β-globin gene cluster provides new insights into mechanisms in developmental gene regulation and network structure in local chromosomal architecture. Furthermore, computational results in mESCs reveal a role for chromatin interacting loops between enhancers and promoters in regulating alternative transcripts of the pluripotency gene OCT4.

## Introduction

In eukaryotic cells, DNA is packaged into chromatin by histones and associated proteins complexes resulting in chromatin through hierarchical architectures [1–3]. The dynamic regulation of chromatin structure underlies many other nuclear processes, including transcription and replication by altering DNA accessibility to the regulatory factors [4, 5]. Chromatin interactions are defined as contacts among chromosomal regions that may be far from each other along primary DNA sequence but close in the 3D space. The multiscale spatial organization of chromatin interactions has been shown to play important roles in many cellular processes [6–9]. Moreover, aberrant chromatin interactions are often linked to human diseases including cancers and are considered as the main contributing factors in disease development [10, 11]. Therefore the identification of the three dimensional chromatin structure is crucial to decipher normal genome functions and how the disordered chromatin interactions are linked to divergent diseases.

Several methods have been established to enable high-throughput analysis of long-range chromatin interactions. Among these, Chromosome Conformation Capture (3C) [12] and its derivatives 4C [13], 5C [14], ChIA-PET [15, 16], Capture-C [17, 18], HiChIP [19] and Hi-C [20, 21] are based on proximity ligation of spatially close chromosomal loci [22]. While these assays have significantly improved our understanding of the spatial and functional organization of genome, distinct limitations have been observed with each method. Specifically, 3C, 4C and 5C-based assays only detect interacting loci with pre-defined PCR primers and oligonucleotide probes [22, 23]. ChIA-PET and HiChIP focus on chromatin interactions mediated by pre-defined protein factors such as RNAPII, CTCF, RAD21 or other transcription factors (TFs) [15, 16, 19, 24]. Hi-C often suffers from low coverage and low resolution for specific genomic regions. As an alternative to 4C, the CAPTURE-3C-seq approach can unbiasedly identify high-resolution and locus-specific long-range DNA interactions by combining 3C and sequence-specific genomic targeting based on biotinylated dCas9 proteins [25, 26].

Here we present C3S, a model-based method and pipeline, for processing raw CAPTURE-3C-seq datasets and determining the statistical significance of the detected chromatin interactions. C3S can automatically define the bin size based on the binding peak of the dCas9 targeted genomic region (bait region). It employs a series of negative binomial distributions for spatially covering the random noise backgrounds of intra- and inter-chromosomal interactions separately, which could be introduced by distance-dependent random polymer ligation and/or dCas9 off-target binding. When applied to CAPTURE-3C-seq data of the human β-globin gene cluster in K562 cells, C3S successfully identified known and new regulatory interactions that were subsequently validated to be required for the proper expression of β-globin genes. Integrative and topological analysis of interacting loci and associated TFs uncovered candidate TFs involved in mediating or maintaining these interactions. We also detected distinct chromatin interacting clusters between enhancers and promoters for variant transcripts of OCT4 gene in mouse ESCs, providing new insights into the role of alternative enhancer-promoter usages in the regulation of transcriptional variants.

## Materials and methods

### C3S workflow

**Fig 1:**
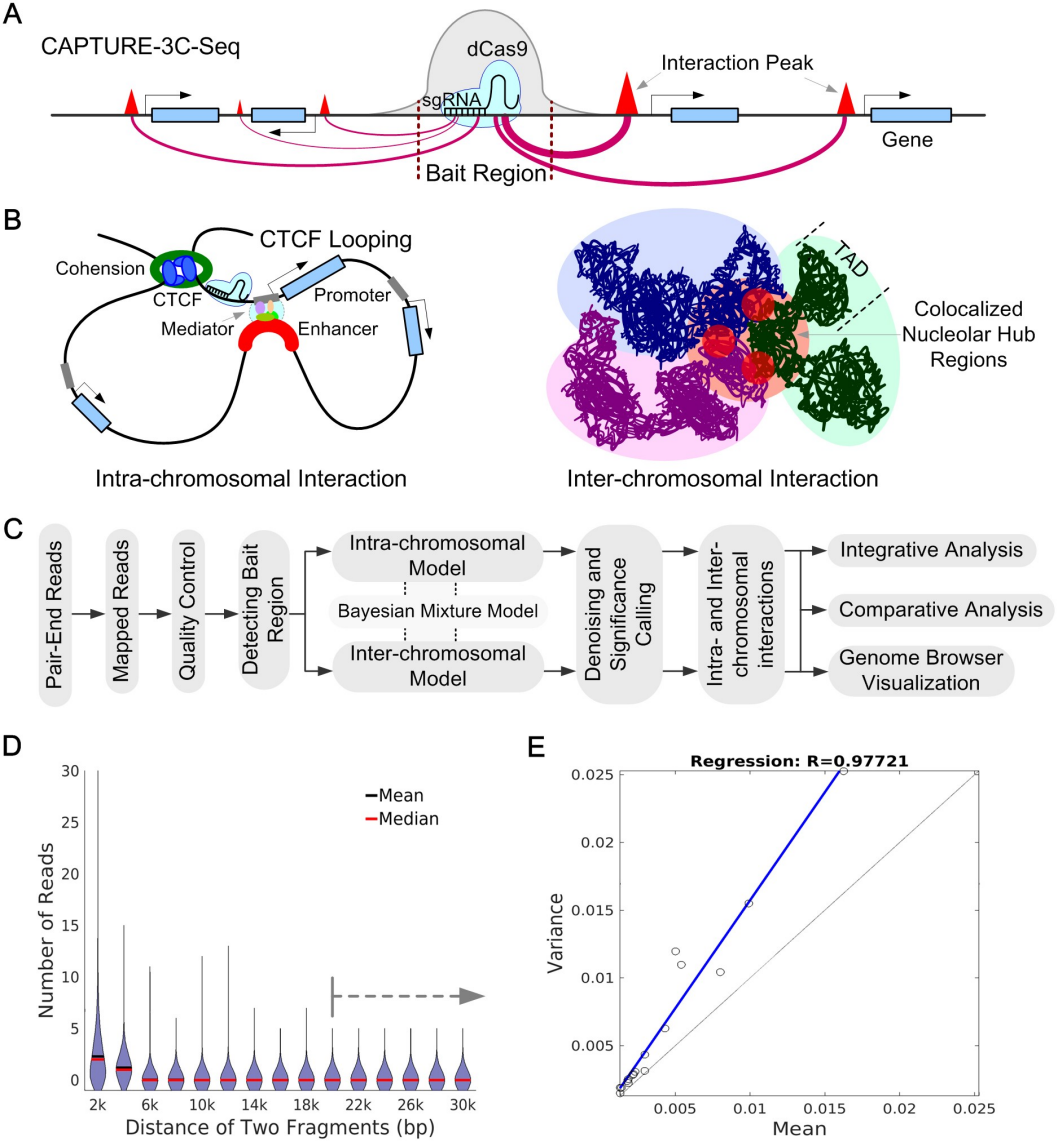
C3S workflow. **A**. A schematic of detecting chromatin interactions by CAPTURE-3C-seq. The biotinylated dCas9-based ChIP-seq was used to define the bait region. The red triangles denote the peak intensities and different red lines indicate long-range interactions associated with different significance scores. **B**. Two typical examples of intra- and inter-chromosomal interactions. **C**. The workflow of C3S pipeline. C3S takes raw reads as input and output multiple types of information for subsequent analysis. Detailed steps can be found in S1 Fig. **D**. Distance-based distributions are observed for random intra-chromosomal interactions. The dotted line shows that the distributions are similar after 20kb distance. **E**. Correlation between means and variances of distributions is shown.

To detect locus-specific long-range chromatin interactions, we developed the CAPTURE-3C-seq method using *in vivo* biotinylated dCas9 and sequence-specific sgRNAs to capture long-range DNA interactions associated with targeted genomic loci (or bait regions) (Fig 1A) [25, 26]. This protocol employs paired-end sequencing reads with one end localized at bait regions and the other end at interacting chromosomal regions. If the other end is at the same chromosome, the paired end indicates an intra-chromosomal interaction, if each end is on different chromosomes, an inter-chromosomal interaction. Intra-chromosomal interactions are important in the identification of CTCF loops and enhancer-promoter interactions [16, 24], while inter-chromosomal interactions may indicate co-localized nuclear hub regions [27–29] (Fig 1B). Considering that intra- and inter-chromosomal interactions are associated with different levels of background noise, we established different Bayesian models to evaluate the significance of intra- or inter-chromosomal interactions. The method we developed consists of an automatic pipeline from raw reads processing to final significance calling (Fig 1C, S1 Fig). The outputted files support visualization and integrative analysis by genome browser.

### Public datasets used

The public datasets of CAPTURE-3C-seq are available at Gene Expression Omnibus (GEO) database with the accession number GSE88817. The ChIP-Seq data was retrieved from the ENCODE data repository site [30]. The genomic view of long-range genome interactions, ChIP-Seq, DNase-Seq and RNA-Seq data were obtained and exhibited by using the WashU EpiGenome Browser [31]. A summary of the used datasets is listed in S1 Table.

### Reads alignment and pairing

Raw pair-end reads of CAPTURE-3C-seq were mapped to human (hg19) or mouse (mm9) genome assembly as single-end reads using Bowtie2 [32] with the default parameters. Considering that some unmapped reads may be generated by proximity ligation after DpnII digestion, unmapped reads were trimmed with DpnII digestion sites GATC and the longer fragment (>20bp) was remapped. In our tested data, we found that 2%~5% of total reads can be remapped after this trimming procedure. All mapped reads were merged before performing quality trimming using MAPQ>30. The mapped reads from two individual mapping files were then paired, and the paired reads with same positions at both ends were considered as PCR duplicates and discarded.

### Define the size of bait region

For data analysis of chromatin interactions, it is important to define the bin size in order to calculate the significance of interactions between two chromosomal loci (bins). Previous studies of 4C, 5C, Hi-C and Capture-C usually used fixed bin sizes including 2kb, 4kb, 10kb, 40kb based on different sequencing depths to define the interacted regions [13, 17]. Since the peak size of dCas9/sgRNA-targeted genomic regions may be variable in different experiments and chromosomal loci due to differences in sgRNAs and/or chromatin accessibility, the fixed bin size may result in inaccurate positioning of bait regions. To avoid the arbitrary assignment of bait regions, we used MACS2 [33] to calculate the bait region as the peak region surrounding the sgRNA target site. Considering that MAC2 may fail to output the binding peaks for some bait regions that have weak dCas9/sgRNA binding signals, we designed a heuristic method to position the peak of bait regions. To do this, we first defined the local background by using the average reads depth of 100kb surrounding region of the sgRNA target site. Starting from the sgRNA target site, we smoothed the reads depth for each base position as the average depth of its up- and downstream 50 bp, and we continually counted the positions containing higher reads depths than local background as the bait region.

### Bayesian mixture model of detecting chromatin interactions

After defining the bait region, we partitioned the chromosomes as bins with the same size as the bait region. We then extracted pair-end sequence tags (PETs) that have distinct genomic locations at one or both ends of the pair-end reads within bait region, and classified the PETs into three categories. 1) PETs with both ends located within the bait region were considered as self-ligated reads and discarded. 2) PETs with only one end located within the bait region were considered as the interactions from bait region to other chromosomal regions. 3) PETs with neither end located within the bait region were considered as random ligations or interactions associated with dCas9 off-target regions and used as the noise background. Considering that the interaction background for random ligations located at the same or different chromosomes may be different, we used two different background models to calculate the significance for intra- and inter-chromosomal interactions.

#### (1) Model for intra-chromosomal interactions

To analyze the significance of intra-chromosomal interactions, we first collected interaction values *X*_*d*_ of any two regions that have distance of *d* times (*d* ≥ 1) of bait region size *l* in the same chromosome (excluding the bait region) as the noise background of intra-chromosomal interactions. For a given distance *dl*, we randomly selected 10000 paired regions from the same chromosome and counted interaction distributions of *X*_*d*_. First, we found that the means of *X*_*d*_ were decreased when distances increased, but the distributions were similar after 20kb distance (Fig 1D). The variances are bigger than means for all different distance-based distributions. Furthermore, linear regression analysis showed that variances are proportional relationships with means (Fig 1E), indicating that *X*_*d*_ could be following a negative binomial distribution (NB), e.g. *X*_*d*_~NB(*r*_*b*_, *p*_*b*_), parameter *r*_*b*_ is the number of unknown failures and *p*_*b*_ is the probability of success. To understand this, we have the probability mass function of NB as ${p(X}_{d})=(\begin{array}{c} X_{d}+r_{b}-1\\ X_{d} \end{array}){\left(1-p_{b}\right)}^{r_{b}}{p_{b}}^{X_{d}}$ and then the variance/mean ratio is $\frac{1}{1-p_{b}}>1$ (given *p*_*b*_ < 1).

Since the distributions were different before 20kb distance but similar after that, we employed a series of Bayesian mixture models to describe interaction background with different distances of *dl* ≤ 20*k*, and one model for paired regions with distances bigger than 20kb. For each negative binomial distribution, we counted interactions among them as the background, and estimated its parameters by using maximum likelihood estimator in Python package ‘statsmodels’. Thus, *X*_*d*_ can be used as the random interaction frequency between any two chromosomal regions (with distance of *dl*). Given the true observation *x*_*d*_ as the number of PETs from the bait region to another chromosomal region with *dl* distance, we calculated P-values to reflect the significance of *x*_*d*_ as *p*(*x*_*d*_) = *p*(*X*_*d*_ < *x*_*d*_). The bigger *p*(*x*_*d*_) indicates lower possibility of random interactions that are bigger than *x*_*d*_, suggesting higher confidence of interactions between the bait region and the chromosomal region. We also used the Bayesian factor (BF) to compare the hypothesis *H*_0_ that specific interactions have occurred between the bait region and a given chromosomal region against the alternative hypothesis *H*_1_, representing no interactions between them. The BF is a strength measure for comparing two hypotheses, providing a natural way to control false discovery rate (FDR). Here, we assigned the prior odds *P*(*H*_1_)/*P*(*H*_0_) as 0.001, indicating that random collision bigger than true interactions is a rare event. Based on previous BF interpretation method [34], interactions with BF more than 20 are considered as high-confidence interactions.

#### (2) Model for inter-chromosomal interactions

For interactions between the bait region and other regions on different chromosomes, we developed the background model by using the random collisions among different chromosomes. Since the spatial organization of chromosomes in cell nuclei may cause different random collisions, we constructed models for the bait-region-localized chromosome (bait chromosome) and each other chromosome. To obtain enough background signals, we first extended the bait region to 1 Mb and split all chromosomes into 1 Mb regions. We randomly selected 10000 region pairs containing one end from the bait chromosome (excluding the bait-region) and the other end from another chromosome. We counted the PET numbers for these region pairs as the background for bait chromosome and the chromosome. The random interactions are also fitted as negative binomial distribution. Thus, we employed chromosomal specified background models for significance calling of inter-chromosomal interactions. We then calculated the P-value and Bayesian factor to determine whether the interactions from the bait region to inter-chromosomal regions were significant.

### Software implementation and data visualization

C3S is implemented using python and shell scripts that can be easily installed on Linux operating system. C3S can be downloaded from https://github.com/YONGCHENUTD/C3S. C3S provides the intra- and inter-chromosomal interactions that can be visualized using WashU EpiGenome Browser [31]. Considering that the interactions generated from CAPTURE-3C-seq are usually sparse, we store the output files as interaction lists. Then the integrative and coordinated analysis can be performed within the same browser.

## Results

### Quality control in Capture-3C-Seq data analysis

**Fig 2:**
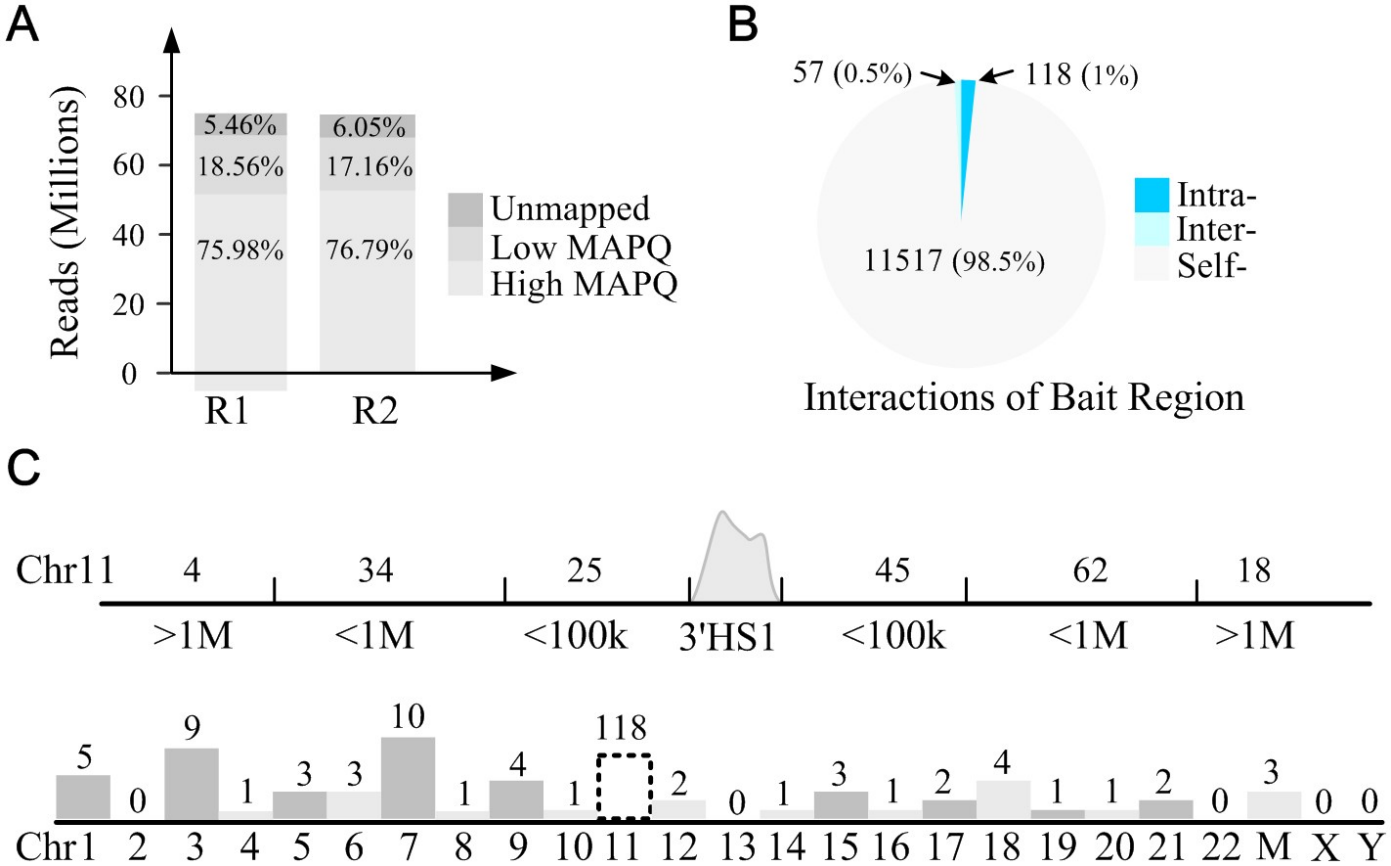
C3S quality controls and basic statistical analysis. **A**. Statistics metrics of read alignment. **B**. The percentages of three types of PETs including intra-chromosomal PETs, inter-chromosomal PETs and self-ligated PETs. The numbers in bracket show the percentages of different PETs among total PETs. **C**. PET count distribution of intra-chromosomal interactions.

We applied C3S to the CAPTURE-3C-seq data of the human β-globin gene clusters containing 5 upstream DNase I hypersensitive sites (HS1-5) at locus control region (LCR) and the linked β-like globin genes. The β-globin gene cluster contains well-defined CTCF-mediated long-range DNA interactions (or looping) between the flanking HS5 and 3’HS1 insulators. We first analyzed the long-range interactions associated with dCas9-captured 3’HS1 insulator. After aligning ~76.5 millions raw reads to the hg19 reference genome independently, we achieved mapping ratios of 89.54% and 88.95% for two independent replicate CAPTURE-3C-seq experiments. After filtering low mapping quality reads, 75.98% and 76.79% reads were kept for subsequent analysis (Fig 2A). To avoid the hard threshold for defining bait region using a fixed bin size of 2kb or 3kb used in Hi-C experiments [13, 17], C3S provides a flexible way to define the bait region. The bait region of 3’HS1 was calculated as 2628bp for the sgRNA targeted genomic region. After defining the size of the bait region, a total of 11692 PETs were classified into three categories, intra-chromosomal PETs, inter-chromosomal PETs and self-ligated PETs. We found 98.5% of PETs to be self-ligated PETs with both ends in the bait region, indicating a high sgRNA targeting efficiency. There are 118 (1%) PETs linking bait region with different regions at the same chromosomes and 57 (0.5%) PETs linking bait region with regions from other chromosomes (Fig 2B). The spatial distributions of PETs are counted for both intra- and inter-chromosomes (Fig 2C). For the 118 intra-chromosomal PETs, we found that they are mainly located within ±1M distance from the bait region (96/118 or 81.36%). Meanwhile, there are 80 PETs located at 3’ downstream and 38 at 5’ upstream (80/38 or 2.11 fold). For the 57 inter-chromosomal PETs, we found they are almost uniformly distributed but with relatively high numbers of PETs for chr3 (9 PETs) and chr7 (10 PETs) (Fig 2C).

### Detecting intra-chromosomal chromatin interactions from local bait regions

After the identification of PETs and bait regions, the C3S pipeline provides a computational framework to determine the significance of interactions between the bait region and any other chromosomal regions. As shown in Fig 1D, the random collisions decrease with the increasing distance of two interacting loci, consistent with previous studies using Hi-C [20] and ChIA-PET [15, 16]. To capture this spatial information, we used a series of negative binomial distributions for different interacting distance of two loci. This strategy is also important considering that enhancer-promoter interactions are usually within tens to hundreds kb within topologically associated domains (TADs) [16, 20]. By calculating the P-values and BFs, we identified the significant interactions for subsequent analysis.

**Fig 3:**
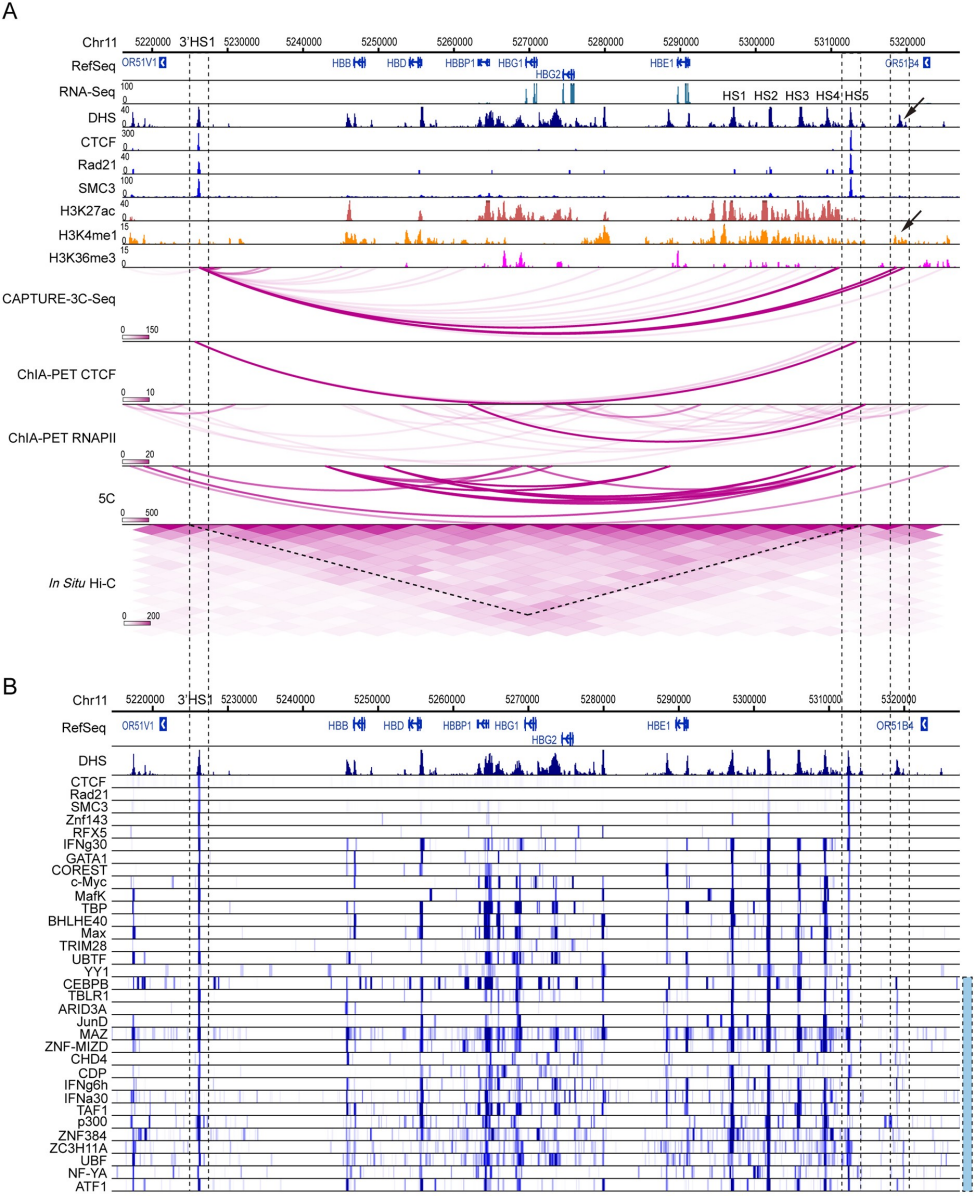
Detecting intra-chromosomal interactions at the 3’HS1 region. **A**. The identified long-range interactions are shown together with DHS, histone markers (H3K27ac, H3K4me1 and H3K36me3), CTCF, RAD21 and SMC3 binding signals from the ENCODE database. Comparisons with 5C, *in situ* Hi-C, RNAPII and CTCF ChIA-PET are shown on the bottom. The arrows indicate the peak signals of DHS and H3K4me1 that co-localize with the interactions at the 3’ region of the OR51B4 gene. **B**. ChIP-seq data of 33 TFs are shown. The right blue bar denotes the 17 TF tracks with strong binding peaks at the 3’ region of the OR51B4 gene (Chr11:5310800–5320000).

To evaluate the performance of C3S in detecting known and new interactions, we analyzed the identified significant interactions (BF>20) associated with the 3’HS1 insulator. In total we achieved 118 intra-chromosomal PETs, most of which are associated with the β-globin gene cluster (62 PETs). Although most of 118 PETs were reported to have low BF scores, there are two regions, the HS5 insulator and 3’ region of the OR51B4 gene, showing significant interactions with the 3’HS1 bait region (Fig 3A). We found that the interactions between 3’HS1 and HS5 regions were also observed by several independent datasets including a strong interaction signal reported by CTCF ChIA-PET analysis [16]. The same interaction signal is also confirmed by 5C and Hi-C experiments (Fig 3A). Based on signals of DHS and ChIP-seq of CTCF, Rad21 and SMC3, we observed strong signals at both HS5 and 3’HS1 regions, consistent with their insulator function. We further searched all the TF ChIP-seq datasets from ENCODE project and identified many TFs associated with both regions (Fig 3B). Thus, the integrative analysis of TF binding signals demonstrates that the interactions between 3’HS1 and HS5 regions are mediated by CTCF and cohesin complexes. We then analyzed the interactions between 3’HS1 and the 3’ region of the OR51B4 gene. Although there is no interaction signal based on CTCF and RNAPII ChIA-PET, the interactions are confirmed by Hi-C analysis (Fig 3A). Moreover, we found that this region is associated with strong DHS and H3K4me1 signals, suggesting a possible *cis*-regulatory element (indicated by arrowheads, Fig 3A) for the human β-globin gene cluster. From over 200 available TF ChIP-seq datasets, we identified 17 TFs co-localizing with the interacting region (blue bar indicated, Fig 3B). These results suggest that our method not only identifies known CTCF-mediated long-range DNA interactions, but also uncovers other interactions with potential regulatory activities.

### Detecting inter-chromosomal interactions

**Fig 4:**
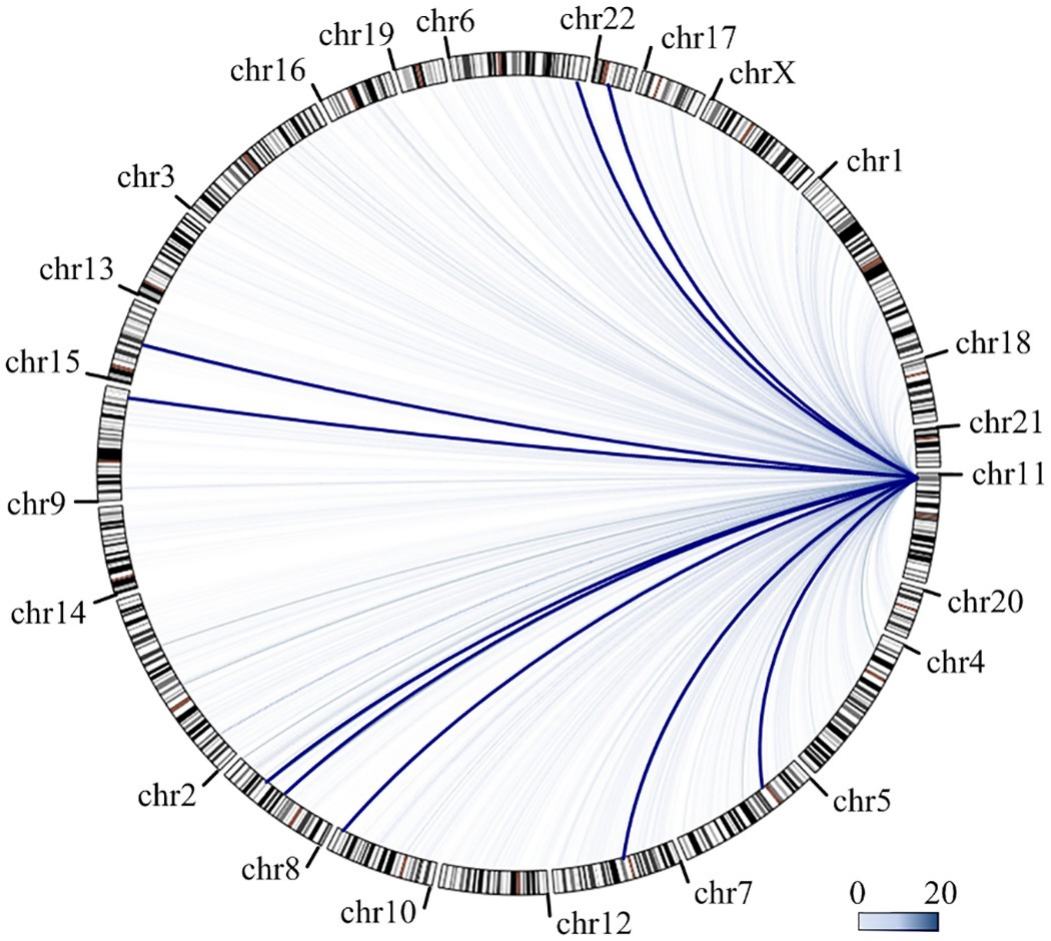
Detecting inter-chromosomal interactions at the β-globin region. The light blue curves indicate nonsignificant interactions, and the bold blue curves indicate 9 interactions with BF>20.

In addition to intra-chromosomal interactions, genomic loci on separate chromosomes are also observed to frequently interact with each other in the nuclear space. Many examples of inter-chromosomal interactions have been confirmed by DNA-FISH [35], SPRITE [27], and validated to be involved in controlling gene expression in normal and diseased cells [36–38]. Often, in previous analyses of 3C, 4C, 5C, ChIA-PET and Hi-C data, the inter-chromosomal reads pairs are often discarded, resulting in absence of inter-chromosomal interactions as represented in ENCODE and Roadmap web servers [31]. Given that the spatial organization of chromosomes in cell nuclei may introduce a different background of random collisions among chromosome pairs, we provide a series of models to detect the significant inter-chromosomal interactions. For each chromosome, we constructed a model for filtering the random interactions between the bait region-containing chromosome and other chromosomes. By combining 9 independent CAPTURE-3C-seq experiments at 9 independent bait regions at the β-globin gene cluster, we obtained a total of 2582 inter-chromosomal interactions between the β-globin region on chr11 and all other chromosomes. Although most of the interactions are uniformly distributed among chromosomes, we detected 9 interacting regions on 8 chromosomes associated with significant interactions with the β-globin gene cluster (BF>20, Fig 4). Interestingly, inter-chromosomal interactions were also observed in EndoC-βH1 cells, in which 6 chromosomes (chr5, chr6, chr7, chr8, chr9 and chr15) were found to be physically interacted with the promoter region of INS gene that is located ~2Mbp from the β-globin cluster [39]. Interactions from chr7 to chr11 were also independently detected in MCF-7, HMEC and MDA-MB-231 using 4C methods [40]. Furthermore, the LCR region of the mouse β-globin gene cluster and CTCF proteins were found to be associated with inter-chromosomal interactions by DNA-FISH and 4C experiments [41, 42]. It is important to note that the existing evidence is only based on 3C-mediated interaction assays or FISH-based imaging, and the functional role of the identified inter-chromosomal interactions in regulating β-globin gene transcription remains unknown. Nonetheless, the C3S method provides opportunities for the identification and statistical analysis of inter-chromosomal interactions as candidate genomic regions for further experimental validations.

### Detecting chromatin interactions across cell models

**Fig 5:**
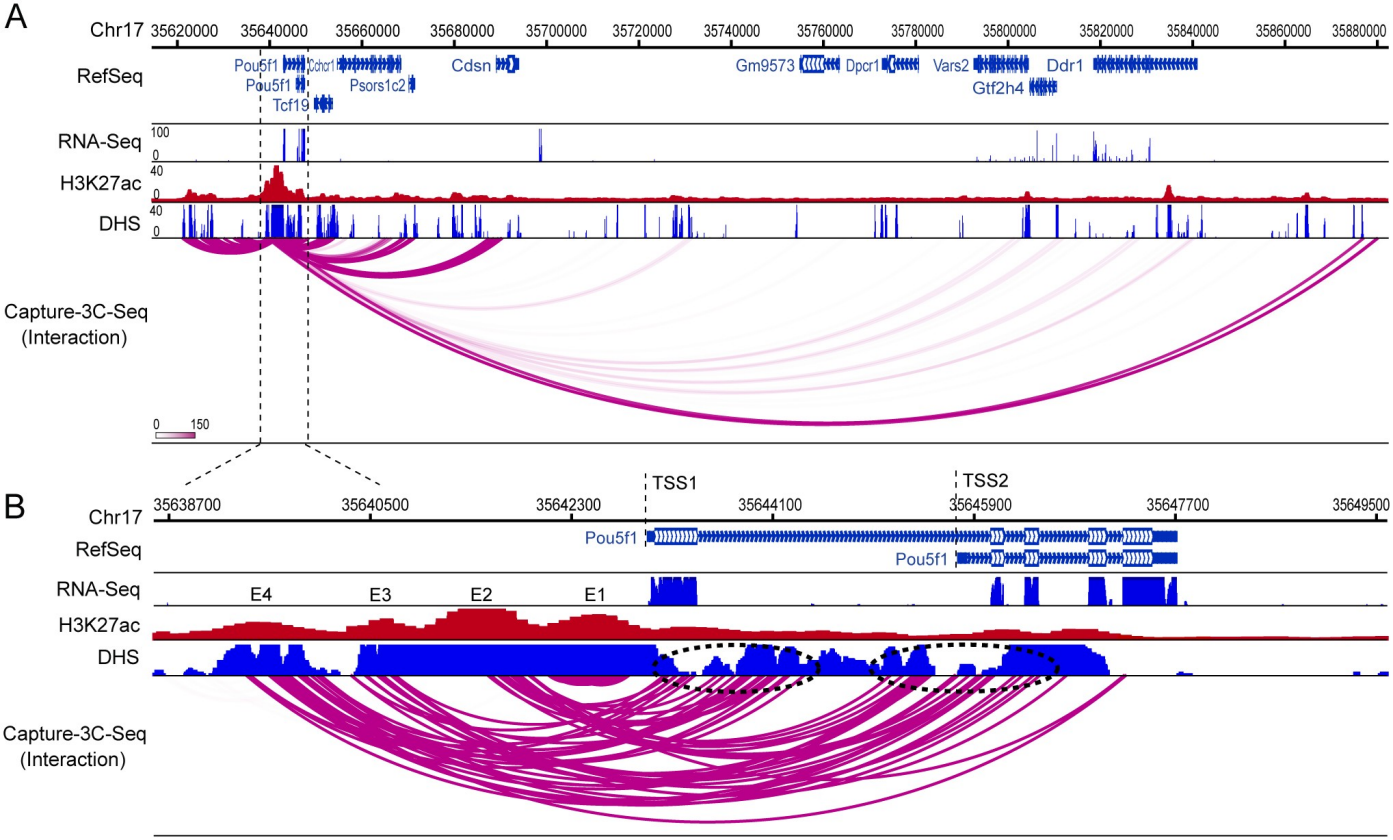
Detecting chromatin interactions associated with the OCT4 super-enhancer in mESCs. **A**. The zoom-out view of the captured chromatin interactions at the OCT4 super-enhancer region (Chr17:35620000–35688000). **B**. The zoom-in view of the captured chromatin interactions at the OCT4 super-enhancer. The dotted ovals show two distinct promoter regions (TSS1 and TSS2) associated with clustered interactions with discrete constituent enhancer (E1 to E4) (Chr17:35638700–35649500).

To demonstrate the performance of C3S across cell models, we analyzed interactions identified by the dCas9-captured super-enhancer for the OCT4 (Pou51) gene in mouse ESCs. OCT4 is a transcription factor that acts as a master regulator of pluripotency in embryonic stem cells. At the 5’ upstream of the OCT4 gene, a super-enhancer was identified to contain 4 constituent enhancers (E1 to E4; Fig 5B); however the interacting promoters and other *cis*-regulatory elements associated with these enhancers remained unclear [43]. By analyzing the CAPTURE-3C-seq data for the OCT4 super-enhancer, C3S successfully outputted many significant long-range interactions between the captured super-enhancer region and other interacting regions including the OCT4 gene as well as other genes including Cchcr1, Psors1c2 and Cdsn in the chromatin neighbourhood (Fig 5A). Additionally, a very long distance interacting region (~240kb) was identified between the 3’ region of this super-enhancer and the 5’ region of the Ddr1 gene, which associated with strong DHS signals.

Interestingly, we observed distinct interactions between individual enhancers and the promoters of two transcript variants for the OCT4 gene (Fig 5B), consistent with two annotated transcripts of mouse OCT4 gene in Refseq database [44, 45]. Importantly, while E3 and E4 interact strongly with both promoters (TSS1 and TSS2), E2 predominantly interacts with the transcript variant 2 promoter (TSS2). These findings suggest that distinct enhancers and their combinations within the same super-enhancer may interact with promoters of different transcript variants to regulate their transcription. Moreover, all the interactions detected at E1 were associated with local bait regions, suggesting that distinct long-range DNA interactions may regulate different constituent enhancers within the same super-enhancer cluster. These findings are reminiscent of our recent reports that a subset of super-enhancers is hierarchically organized containing individual constituent enhancers associated with distinct chromatin interactions and/or function [46, 47].

### Integrative and topological analysis to understand functional genome organization

**Fig 6:**
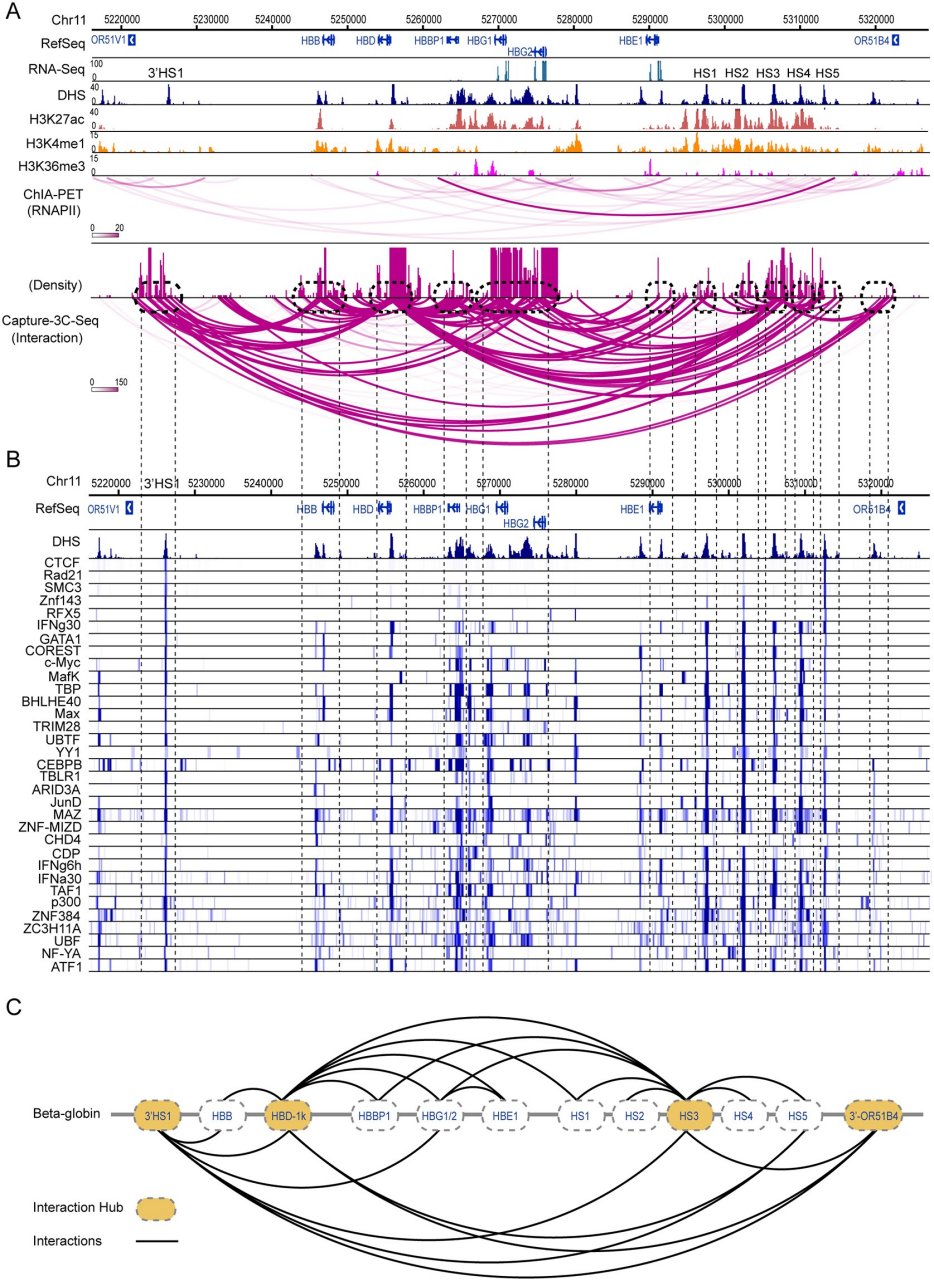
Integrative and topological analysis of long-range interactions and TF binding at the human β-globin gene cluster. **A**. Integrative analysis the β-globin gene cluster by histone modifications and captured long-range DNA interactions. **B**. Potential TFs with ChIP-seq binding signals overlapping with the interacting genomic loci at the β-globin gene cluster. **C**. Topological plotting of the chromatin architecture at the β-globin gene cluster.

By focusing on the β-globin gene cluster, we performed 9 independent CAPTURE-3C-seq experiments to capture chromatin interactions associated with discrete *cis*-regulatory elements including gene proximal promoters, distal enhancers, insulators, and other functional regions (S1 Table). Using C3S on these datasets, we detected a number of significant interactions associated with one or multiple captured elements. We next performed topological and integrative analysis of all the interacting profiles associated with the β-globin gene cluster. First, we merged all the interactions from 9 CAPTURE-3C-seq experiments and identified 12 genomic loci containing strong long-range chromatin interactions and enrichment of TF binding peaks (Fig 6A and 6B). At the flanking regions, 3’HS1 is connected with the HS5 at LCR by a CTCF loop, consistent with an insulated neighbourhood [5]. We observed that the interactions associated with the HBD-1k intergenic region can be classified into two directions, which topologically separate the β-globin gene cluster into two subgroups. The first subgroup consists of the HBB and HBD genes, whereas the second subgroup includes the HBG1, HBG2 and HBE genes. In K562 cells, the HBB and HBD genes are repressed, whereas HBG1, HBG2 and HBE are highly expressed. Our results suggest that the HBG-HBD intergenic region may serve as a major chromatin structural point in controlling expression of activated and repressed β-like globin genes. Second, we performed integrative analysis of the interacting regions with TF ChIP-seq datasets in ENCODE database. We found that the 12 interacting loci highly overlap with binding peaks of 33 TFs (Fig 6B), where the chromatin structural proteins CTCF, Rad21, SMC3, Znf143 and RFX5 are mainly associated with two insulators 3’HS1 and HS5. We also observed that the 3’HS1, HBD-1k, HS3 and 3’ region of the OR51B4 gene are associated with more abundant chromatin interactions than other loci, suggesting that they may serve as the major interacting hubs at the β-globin gene cluster (Fig 6C). Importantly, we observed that some interactions are highly consistent with the interactions detected by the RNAPII ChIA-PET analysis, suggesting that they may be RNAPII-mediated interactions. However, many interactions associated with 3’HS1, HBD-1k, HS3 and 3’ OR51B4 do not overlap with RNAPII-mediated interactions, indicating that CTCF or other unknown TFs may be involved in maintaining chromosomal architectures at these loci. Our results showcase that the C3S software can process the CAPTURE-3C-seq data for integrative and topological analysis of chromatin structure at the captured genomic loci. These results not only provide new insights into the transcriptional regulation of complex gene clusters, but also identify de novo DNA interactions and associated *cis*-elements for functional follow-up studies.

## Discussion

Systematic analysis of genome scale and locus-specific long-range chromatin interactions is critical for understanding chromosomal architectures in gene regulation. With the availability of 3C, 4C, 5C, ChIA-PET, HiChIP, Capture-C, Hi-C and CAPTURE-3C-seq datasets in various model systems, it is of great importance to develop improved computational models for data analysis and integration. C3S is a model-based method for processing the raw CAPTURE-3C-seq data, defining the size of bait region, and determining the statistical significance of the chromatin interactions. It provides quality control at different steps and several output formats for visualization and statistical analysis. By using a series of models, C3S can also process spatial and spectrum signals for noise filtering and detect high-confidence chromatin interactions at multiple scales.

C3S was successfully applied to CAPTURE-3C-seq data of the β-globin gene cluster in human K562 cells and the OCT4 super-enhancer in mouse ESCs. Known and new regulatory interactions were identified to be associated with the regulation of various globin genes and the local chromatin structures. By integrative analysis of TF binding profiles from ChIP-seq studies, we identified 33 TFs that are associated with 12 major interacting loci at the β-like globin gene cluster. Furthermore, C3S has the ability to analyze inter-chromosomal interactions that are usually discarded in 3D genome analyses. Although most of the inter-chromosomal contacts appear to be random interactions, there are several loci associated with significant interactions with the β-globin cluster. Many of the identified interactions at the β-globin *cis*-regulatory elements are either supported by independent RNAPII ChIA-PET analysis, or by multiple ChIP-seq data such as CTCF, Rad21 and SMC3. These findings suggest that the locus-specific chromatin interactions may be classified into structural and regulatory interactions. By applying C3S to the OCT4 super-enhancer-associated interactions in mESCs, we identified distinct interactions between individual enhancers and promoters of different transcriptional variants. These findings indicate that distinct chromatin interactions and/or function are associated with different or hierarchical enhancer organization of a super enhancer. Therefore, the systematic analysis of locus-specific chromatin interactions provides new insights into the underlying principles governing 3D genome organization. Our results establish C3S as an efficient method for processing CAPTURE-3C-seq data across different cell types to elucidate the hierarchical organization of locus-specific chromatin interactions in mammalian genomes.

## Supporting information

S1 Fig Detailed pipeline steps of CAPTURE-3C-seq analysis. The output data files and the processing steps are marked as light green and white respectively.
(TIF)

S1 Table Genomic datasets of CAPTURE-3C-seq and ChIP-seq for K562 and mESCs used in this study. (XLSX)
