# Supplementary figures and images for: Model-based analysis of chromatin interactions from dCas9-Based CAPTURE-3C-seq
Source: PLoS One. 2020 Jul 31;15(7):e0236666. doi: 10.1371/journal.pone.0236666 (PMC7394367; doi:10.1371/journal.pone.0236666)

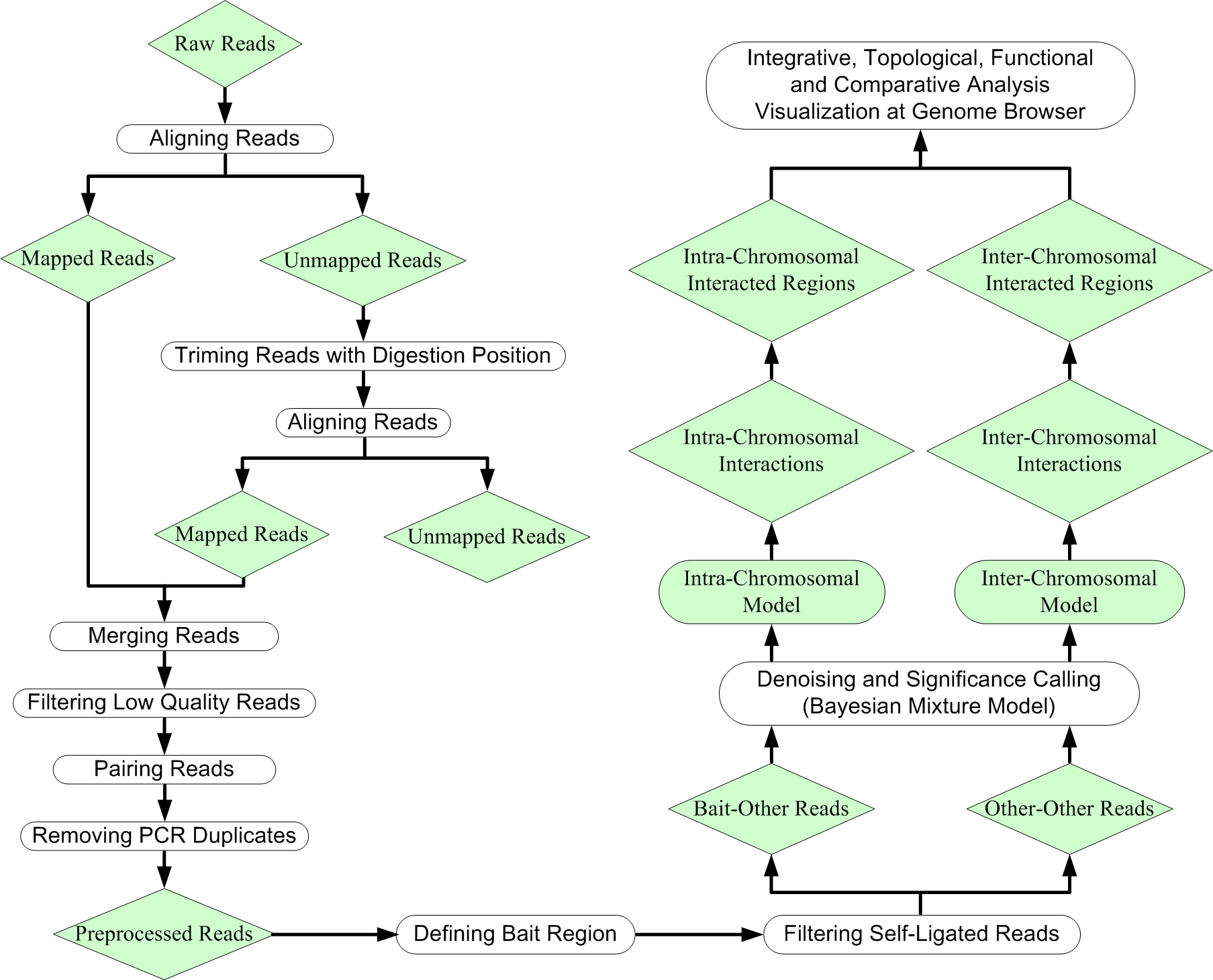

Supplement: S1 Fig — The output data files and the processing steps are marked as light green and white respectively. (TIF) [file pone.0236666.s001.tif]
